# Air pollutant particulate matter 2.5 induces dry eye syndrome in mice

**DOI:** 10.1038/s41598-018-36181-x

**Published:** 2018-12-13

**Authors:** Gang Tan, Juan Li, Qichen Yang, Anhua Wu, Dong-Yi Qu, Yahong Wang, Lei Ye, Jing Bao, Yi Shao

**Affiliations:** 1 Department of Ophthalmology, the First Affiliated Hospital of Nanchang University, Nanchang, 330006 Jiangxi Province China; 2 Department of Ophthalmology, the First Affiliated Hospital of University of South China, Hengyan, 421001 Hunan Province China; 3 Department of Ophthalmology, the Fourth Hospital of Xi ‘an, Xi’an, 710004 Shanxi Province China; 4 Eye Institute of Xiamen University, Xiamen, Fujian 361102 China; 5 Department of ophthalmology, Haidian maternal & child health hospital, Beijing, 100080 China; 6 Environmental Monitoring Station of Xi’an City, Xi’an, 710054 Shaanxi Province China

## Abstract

In this study, we explored the effects of particulate matter 2.5 (PM_**2**.**5**_) eye drops on the ocular surface structure and tear function in mice and established a novel animal model for dry eye research. We found that, following treatment with PM_2.5_, the tear volume and, the tear film break-up time showed statistical differences at each time point (P < 0.05). The FL score of the PM_2.5_-treated group was higher than that of others (P < 0.05). The average number of corneal epithelial layer cells in groups A and B was significantly lower than that in group C (P < 0.05). Scanning electron microscopy and transmission electron microscopy revealed that the number of corneal epithelial microvilli and corneal desmosomes was drastically reduced in group C. PM_2.5_ induced apoptosis in the corneal superficial and basal epithelium and led to abnormal differentiation and proliferation of the ocular surface with higher expression levels of Ki67 and a reduced number of goblet cells in the conjunctival fornix in group C. PM_2.5_ significantly increased the levels of TNF-α, NF-κB p65 (phospho S536), and NF-κB in the cornea. Thus, the topical administration of PM_2.5_ in mice induces ocular surface changes that are similar to those of dry eye in humans, representing a novel model of dry eye.

## Introduction

Dry eye syndrome is an eye disease of abnormal quality or quantity of tears induced by the instability of tear film and ocular surface damage. It is one of the most common ocular surface diseases. The symptoms of dry eye include eye dryness, redness, itching, and severe pain. It can further develop into corneal ulcers, decreased eyesight and even blindness^1^. In addition to impacting eye function, dry eye also affects patients’ daily activities, psychology, and work in varying degrees; hence, it reduces the quality of life. At present, most people believe dry eye is a type of inflammatory immune disease. The instability of tears can be caused by different mechanisms, causing ocular surface damage in dry eye patients^2^. The increased tear osmotic pressure caused by excessive evaporation of tears on the ocular surface is considered one of the contributors as well. When tear film loses stability, it cannot play the role of protecting the cornea, which leads to a cascade of inflammatory reactions in corneal epithelial cells. Inflammatory reactions produce TNF-α, IL-6, and other inflammatory factors, which induce the apoptosis of goblet cells and decrease mucin secretion, thereby affecting the stability of tear film^3^. However, the instability of the tear film further aggravates the tear osmotic pressure, leading to a vicious circle. There are many causes of dry eye, mainly age, systemic disease (such as diabetes and dry eye syndrome)^4,5^, and especially the influence of environmental factors^6,7^. While environmental factors certainly have a great (and underappreciated) influence on tear film stability and ocular surface health, tear film deficiencies (due to genetic factors, age, sex, hormone imbalances, environmental factors, contact lens wear, preservatives, and eye surgery) are the main initiators of this multifactorial disorder.

Environmental pollution has attracted increasing attention, and many studies have shown that environmental pollution can impair human health. PM_2.5_ is one of the indicators to evaluate the severity of environmental air pollution. PM_2.5_ refers to the total atmospheric suspended particles in atmospheric air dynamics with a diameter less than 2.5 μm^8^. It is mainly derived from the burning of fuels (such as motor vehicle exhaustion and coal combustion)^9^ and has a very complex composition, including a large amount of organic matter (such as benzopyrene and polycyclic aromatic hydrocarbons), as well as numerous inorganic components such as sulfates, nitrates, and heavy metals (such as lead and nickel)^10^. PM_2.5_ is not only harmful to the respiratory and cardiovascular systems, causing asthma, thrombosis, and myocardial ischemia, but also shortens the average life expectancy of individuals. However, there are few studies concerning the influence of PM_2.5_ on eyes. Since the eye is one of the organs in direct contact with the outside world, PM_2.5_ might have a direct impact. An investigation of 71 drivers by Torricelli *et al*. reported that the BUT values for these drivers were lower than that in a normal person^11^. Tatsuya *et al*. surveyed patients with acute conjunctivitis from May to October 2012 and found that the number of patients with acute conjunctivitis was increased with a higher level of PM_2.5_^12^. Camara *et al*. investigated the influence of volcanic smoke. The main components of these pollutants are PM_2.5_, which have been found to cause some ocular symptoms, such as eye itching, foreign body sensation, tears and burning, as well as some other signs such as conjunctival congestion, increased mucus secretion, conjunctival keratoconus, swelling of the eyelids and conjunctival edema^13^. Although PM_2.5_ can cause many symptoms, the mechanism by which PM_2.5_ causes damage is unclear.

## Materials and Methods

### Corneal Epithelial Cell Culture

The simian virus-40 (SV40)-transformed HCE cell line (RIKEN Biosource Center, Tokyo, Japan) was cultured in Dulbecco’s modified Eagle’s medium (DMEM) (F-12; Invitrogen) supplemented with 6% fetal bovine serum, 7.5 mg/mL insulin, and 10 ng/mL epidermal growth factor^14^.

### Cell counting kit-8 (CCK-8) assay

HCECs (5 × 10^3^) were treated with 10 μg/mL, 50 μg/mL, 100 μg/mL, 250 μg/mL, 500 μg/mL, 750 μg/mL, or 1000 μg/mL PM_2.5_, and the culture medium was used as the control. The cell counting kit-8 (CCK-8) assay (Tokyo, Japan) was used to assess cell proliferation as previously described^15^.

### Wound closure assay for assessing migration

The wound closure assay was used to measure the migration of HCECs as previously reported^16^. Briefly, a 0.6-mm-width uniform scratch wound was created in a confluent HCEC monolayer using a pipette tip. The cells were further incubated in the presence of 1% BSA or 5.0 mg/mL PM_2.5_ for 0, 8, and 12 hours.

### Phagocytosis procedure

Latex bead (LB) solution (Sigma L4530, carboxylate modified polystyrene, USA) was prepared at a ratio of 1:10 in HCE culture medium. HCE cells were plated at 1 × 10^5^ cells per well in 24-well culture plates and grown until 80% confluency prior to treatment. The culture medium was removed and replaced with culture medium supplemented with LB solution. Next, the cells were washed and mounted in mounting medium containing 4, 6-diamino-2-phenyl indole (DAPI, Burlingame, CA, USA) at 0, 3, 6, 9, 12, 24, or 48 hours after treatment. Images were photographed with a Nikon TE-2000 U Eclipse epifluorescence microscope (Nikon Instruments, Tokyo, Japan).

### Animal preparation

In total, 90 male specific pathogen-free (SPF) BALB/c mice (18–21 g in weight, from Laboratory Animal Center of Xi’an Jiao Tong University College of Medicine, Xi’an, China) were used in this study. No abnormality was found in the anterior segment and fundus when examined using a slit lamp microscope. The results for the Schirmer I test (SIT) were ≥10 mm/5 min. The mice were housed in a standard environment throughout the study^17^. All procedures were performed in accordance with the ARVO Statement for the Use of Animals in Ophthalmic and Vision Research and were approved by the animal ethics committee of Xi’an Jiao Tong University College of Medicine (Xi’an, China).

### Acquisition of PM_2.5_ and preparation of eye drops

PM_2.5_ samples were provided by the Xi’an environmental monitoring station. During October 1 to 31, 2015, a super station at Xi’an City acquired PM samples with sizes of 2.5 μm using the TH-16A four-channel atmospheric particulate automatic sampler (Wuhan Tianhong Instrument Ltd) and filtered them through Whatman PTFE membranes. Sampling was conducted continuously for 22 hours a day from 10:30 am to 8:30 am the next day. PTFE membranes containing PM_2.5_ were cut into 1-cm × 1-cm pieces, immersed in distilled water and oscillated ultrasonically for 45 min for 3 times. After 6 layers of gauze filtration, the samples were vacuum freeze-dried and weighed^18^. The samples were then stored at 4 °C. For the preparation of PM_2.5_ eye drops, PM_2.5_ samples were diluted in sterile PBS to form a concentration of 5 mg/mL and then were vortexed ultrasonically. The preservative benzyl bromide was added to two groups of eye drops (PM_2.5_ and PBS) with the concentration controlled at 0.005%. The eye drops were kept at 4 °C.

### Animal experimental procedure

Ninety mice were divided into three groups randomly (n = 30). The right eyes of each group were treated with the following substances 4 times daily: Group A, negative control (NC); Group B, PBS; Group C, 5.0 mg/ml PM_2.5_. The frequency and doses of PM were determined previously in a pilot experiment. Before treatment, all the mice were confirmed to be free of any ocular diseases. The Schirmer test, fluorescein staining, the tear film break-up time (BUT) test, the inflammatory index, and H&E staining were performed sequentially before and 4, 7, and 14 days after treatment. All the mice were euthanized on day 14, and the eyes were harvested for histological analysis and western blotting.

### Tear volume, Fluorescein, and BUT measurement

The phenol red thread tear test with phenol red-impregnated cotton threads (FCI Ophthalmics, Pembrooke, MA, USA) was used to measure the volume of tears on days 0, 4, 7, and 14 post-treatment. The tear volume, fluorescein and BUT were measured as previously described^19,20^. The average of three measurements of each eye was considered as the final readout^21^. The fluorescein score was analyzed as follows: 0, absent; 1, slightly punctate staining less than 30 spots; 2, punctate staining more than 30 spots, but not diffuse; 3, severe diffuse staining but no positive plaques; 4, positive fluorescein plaques.

### Evaluation of inflammation

The inflammatory response was visualized using a slit lamp on days 0, 4, 7, and 14 post-treatment, and the inflammatory indices were evaluated as previously described^22^. Briefly, the inflammatory index was calculated as the sum of the scores of the following parameters divided by 9: the thickness of the ciliary hyperemia (0: absent; 1: less than 1 mm; 2: 1 to 2 mm; 3: more than 2 mm); the presence of central corneal edema (0: absent; 1: present with visible iris details; 2: present without visible iris details; 3: present without visible pupil); and the presence of the peripheral corneal edema (0: absent; 1: present with visible iris details; 2: present without visible iris details; 3: present with no visible iris).

### Periodic Acid Schiff (PAS) and Hematoxylin and Eosin Staining

The entire globe, including the superior and inferior forniceal conjunctiva, was enucleated and fixed in formalin. Tissue sections of 4-μm thickness through the superior and inferior conjunctival fornices were stained with PAS (Sigma-Aldrich, St Louis, MO) and hematoxylin and eosin (H&E). Three representative slices from homologous positions of each sample were selected (five samples for each group). Goblet cell density was determined by counting PAS-positive cells in four different sections of each animal and taking the average^23^.

### Terminal deoxynucleotidyl transferase-mediated dUTP biotin nick end labeling (TUNEL)

A TUNEL assay (KeyGen Biotech, China, Nanjing) was performed according to a modification of a published method^24^. Sections stained without biotinylated dUTP were used as negative controls.

### Immunofluorescent staining of Ki67

Immunodetection of Ki67 was performed as described previously^25^. Mouse anti-mouse Ki67 antibody (Abcam, ab16667, Cambridge, MA) was used at a 1:150 dilution as the primary antibody, followed by incubation with ALEXA fluorophore-conjugated secondary antibodies (Invitrogen, USA) and counterstaining with Hoechst 33342 dye (0.5 g/mL, Invitrogen, USA). Images were obtained using a fluorescence microscope (Zeiss, Germany).

### Scanning Electron Microscopy (SEM) and Transmission Electron Microscopy (TEM)

For SEM, the corneas and conjunctiva on day 14 were fixed in 2.5% glutaraldehyde in 0.1 M phosphate buffer (pH 7.4) for 24 h at 4 °C. For TEM, the right corneas were harvested and fixed for 2 h in 2.5% glutaraldehyde and 4% paraformaldehyde in PBS (pH = 7.4). The samples were processed as previously described^26^. SEM images were acquired with a scanning electronic microscope (JSM-6330F, JEOL, Japan), while TEM images were acquired with a TEM microscope (JEM2100HC; JEOL, Tokyo, Japan)^27^.

### Western blotting

The cornea and conjunctiva were lysed with cold RIPA buffer and were subjected to electrophoresis on an 8% SDS-PAGE gel. The following primary antibodies were used: TNF-α (1:400; Abcam ab183218, Cambridge, MA), NF-κB p65 (phospho S536) (1:1000; Abcam ab86299, Cambridge, MA), NF-κB (1:800; Abcam ab16502, Cambridge, MA) and β-actin (1:10,000; Sigma, St. Louis, MO) was used as a loading control^28^. HRP-conjugated goat anti-rabbit IgG (1:10,000; Bio-Rad, Hercules, CA) was used as the secondary antibody. Signals were developed with enhanced chemiluminescence reagents.

### Apoptosis of lacrimal glands

The entire lacrimal glands were dissected and fixed in formalin. Tissue sections of 4-μm thickness were stained using H&E staining and the TUNEL assay.

### Image processing and Statistical analysis

Images were processed using Image-Pro Plus 6.0 software (Graphpad Prism, Inc., La Jolla, CA, USA). One-way ANOVA analysis and post hoc analysis were performed for comparisons between groups using SPSS 16.0.0 (SPSS, Chicago, IL). P < 0.05 was considered statistically significant. Data were represented as the mean ± the standard error.

## Results

### Effect of PM_2.5_ on the proliferation and migration of HCECs

**Figure 1:**
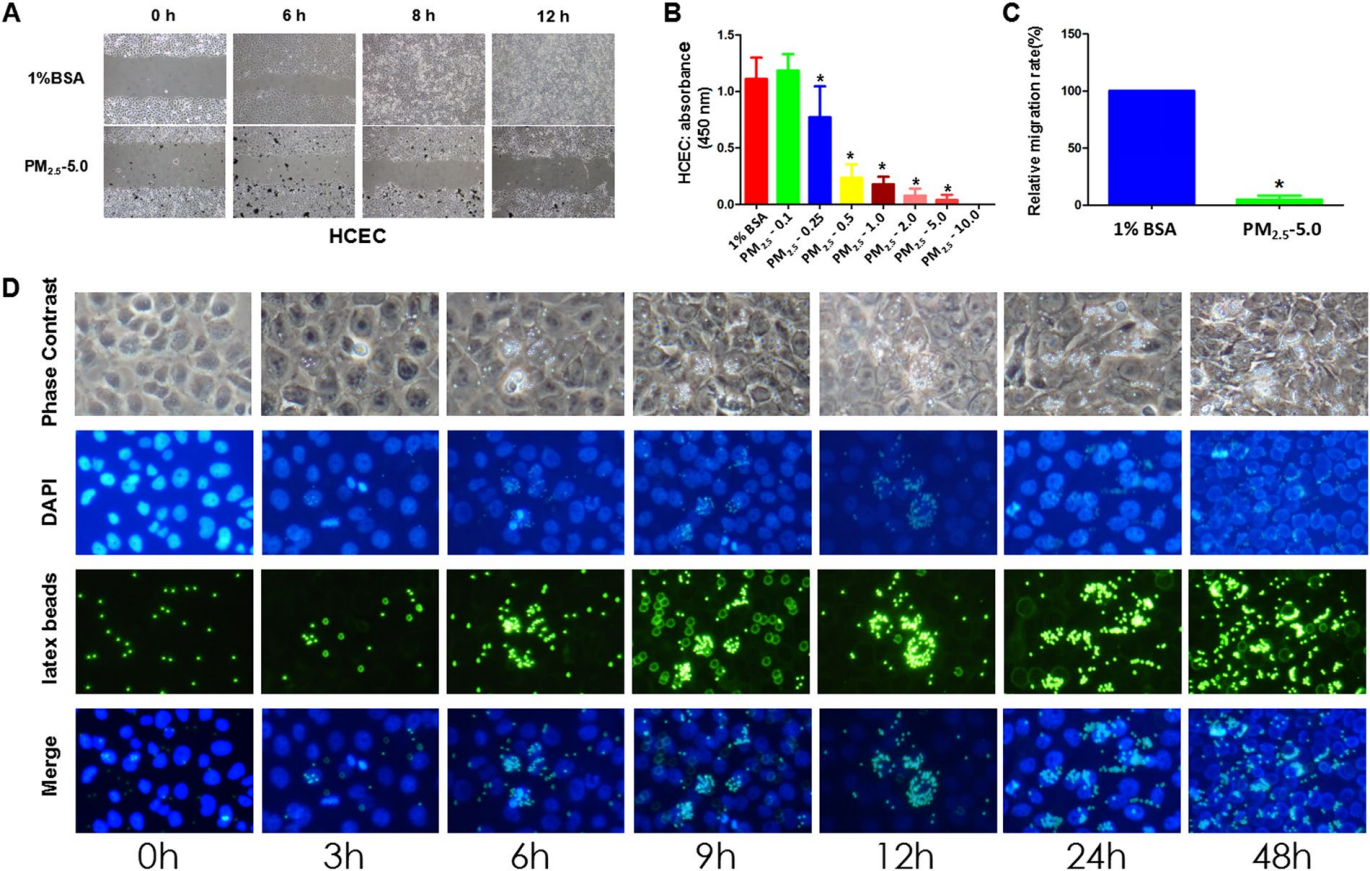
Exposure to different concentrations of PM_2.5_ leads to changes in HCEC proliferation and migration. Notes: (**A**,**C**) 1% BSA and 5.0 mg/ml PM_2.5_ were added to the HCEC culture after scratching; images of wound healing were recorded at 6 hours (h), 8 h, and 32 h. The relative migration rate of HCECs manifested an obvious decline in the PM_2.5_ group compared with that in the culture system supplemented with 1% BSA. (**B**) After treatment with different concentrations of PM_2.5_ (0.1 mg/ml, 0.25 mg/ml, 0.5 mg/ml, 1.0 mg/ml, 2.0 mg/ml, 5.0 mg/ml, or 10.0 mg/ml) for 8 and 12 hours, the growth of HCECs was assessed by the CCK-8 assay. The lowest value of the cell proliferative capacity was detected in the group with 5.0 mg/ml PM_2.5_. (**D**) Uptake of latex beads into HCECs. HCECs were treated with fluorescence-labeled latex beads (shown in green) for 3 h, 6 h, 9 h, 12 h, 24 h, and 48 h. Cell nuclei were stained with DAPI (shown in blue). The amount of latex bead intracellular intake was time dependent and the beads accumulated near the nucleus of HCECs. Each value represents the mean ± the SD, n = 3. *P < 0.05 vs. 1% BSA.

CCK-8 assays were used to measure cell viability at different dosages of PM_2.5_ (0.1 mg/ml, 0.25 mg/ml, 0.5 mg/ml, 1.0 mg/ml, 2.0 mg/ml, 5.0 mg/ml or 10.0 mg/ml). There was no difference between the viability of the HCECs treated with 1% BSA and 0.1 mg/ml PM_2.5_ (P > 0.05). However, PM_2.5_ was shown to reduce HCEC viability with increased concentrations compared with samples treated with 1% BSA (P < 0.05, Fig. 1B). In addition, we evaluated HCEC migration when exposed to 1% BSA or 5.0 mg/ml PM_2.5_ using the wound scratch method. Both groups showed incomplete healing after 8 hours, whereas cells treated with 5.0 mg/ml PM_2.5_ showed less wound healing than cells treated with 1% BSA. At 12 hours after scratching, we found that the migration rate of the HCECs was significantly reduced in the 5.0 mg/ml PM_2.5_-treated group compared with that in the 1% BSA-treated group (P < 0.05, Fig. 1A,C).

### Efficiency of phagocytic uptake in HCECs

To investigate the efficiency of phagocytic uptake in HCECs, we incubated HCECs with fluorescently labeled latex beads (2.5 µm). The beads were internalized by HCECs and were accumulated at perinuclear regions. We observed that HCECs incubated with LB exhibited fluorescent signals within 10 min of incubation, and more fluorescent signals were concentrated near the nucleus with the extension of incubation (Fig. 1D).

### Physiological conditions of treated mice

**Figure 2:**
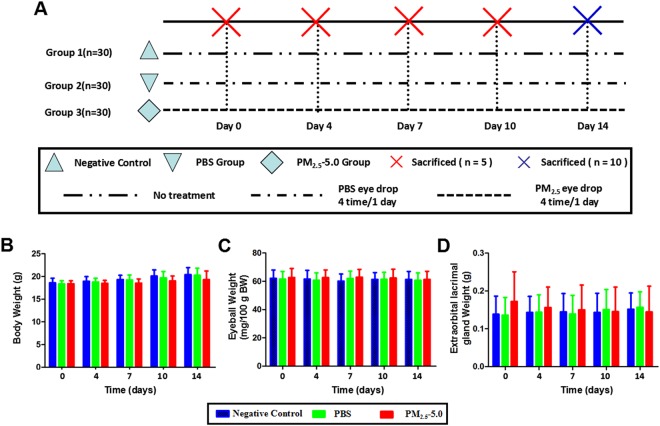
(**A**) Experimental design. Ninety mice were randomized into three groups (n = 30), Group 1: Negative control; Group 2: PBS eye drops, 4 times every day; Group 3: 5.0 mg/ml PM_2.5_, 4 times every day. (**B**-**D**) Evaluation of the body weight (**B**), eyeball weight (**C**), and extra-orbital lacrimal gland weight (**D**) of the mice used in this study. The body weights, eyeball weights, and lacrimal gland weights of the three groups were not significantly different (P > 0.05). Abbreviations: SD, standard deviation; PBS, phosphate-buffered saline; BW, body weight. Each value represents the mean ± the SD, n = 5. These measures were compared with group 1 at the same time points: *P < 0.05 vs. control, ^v^P < 0.05 vs. PBS.

Body weights, eyeball weights, and extra-orbital lacrimal gland weights were measured in all groups at 0, 4, 7, 10, and 14 days after inducing dry eye with PM_2.5_ (Fig. 2A). No significant differences were observed in the body weight, eyeball weight, and lacrimal gland weight among the negative control, 1% BSA and 5.0 mg/ml of PM_2.5_ groups (P > 0.05, Fig. 2B–D).

### Effects of PM_2.5_ on the ocular surface

The inflammatory effects of PM_2.5_ or PBS (4 times/day, daily) on the ocular surface within 14 days were evaluated using inflammation scoring, the stability of tear film, and ocular surface PAS and H&E staining. Based on the preliminary pilot experiments, topical application of PM_2.5_ at 5.0 mg/ml 4 times per day for 14 days was determined as the optimal procedure for the induction of dry eye syndrome in the BALB/c mice. Severe ocular surface damage, ulceration, epithelial defects, and neovascularization were observed with higher concentrations of PM_2.5_ (10.0 mg/ml), while no obvious effects were observed for PM_2.5_ at 5.0 mg/ml (data not shown).

### Stability of tear film and epithelial damage

**Figure 3:**
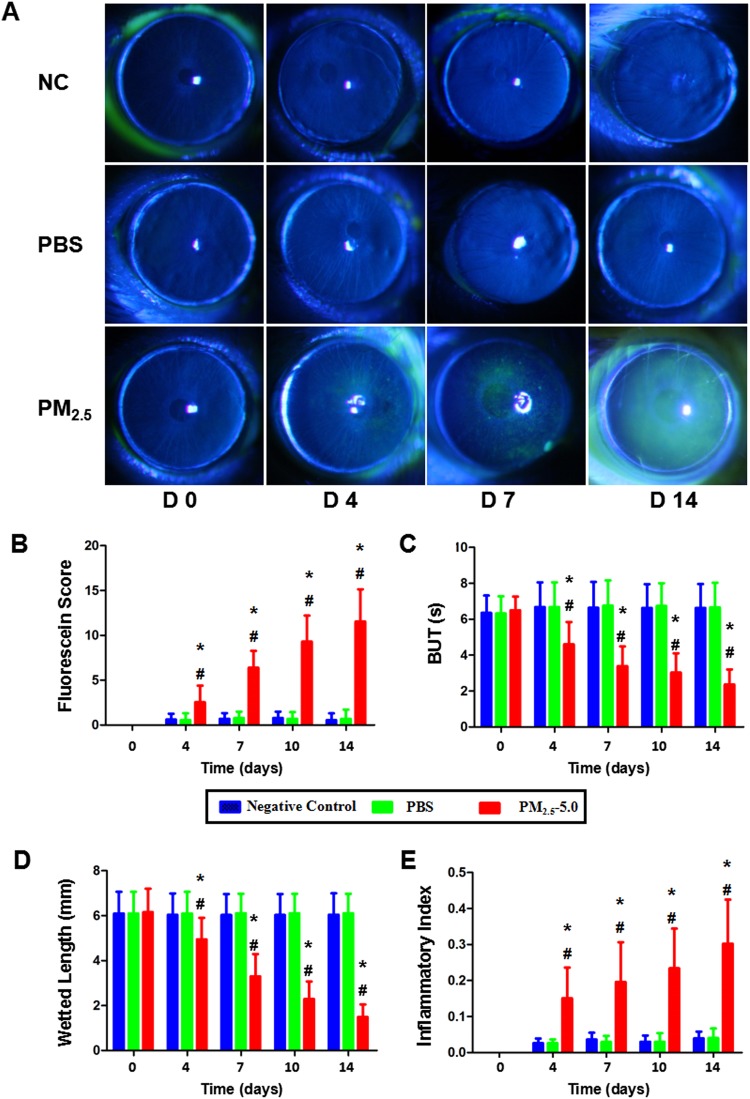
Alterations of the ocular surface and degrees of inflammation after PM_2.5_ treatment. (**A**) Representative examples for day 14 of fluorescein sodium in the PM_2.5_ group (**A**, the lower row of images) compared with the NC and PBS groups (**A**, upper row and middle row of images). Increased corneal fluorescein staining scores and decreased BUTs of the ocular surface were recorded in the PM_2.5_ group at all time points (P < 0.05, respectively, **B**,**C**). Compared with the NC and PBS groups, the phenol red thread test showed markedly decreased tear volume production in the PM_2.5_ group for 14 days of treatment (**D**). (**E**) Increased corneal inflammatory index after PM_2.5_ treatment. Each value represents the mean ± the SD, n = 5. *P < 0.05 vs. control, ^v^P < 0.05 vs. PBS.

Before treatment, no significant differences were observed in BUTs and tear film/epithelial damage scores among the three groups. At day 14, the PM_2.5_-treated group showed significantly decreased BUTs compared with that in the PBS-treated group (^*^P < 0.05 vs. control, ^v^P < 0.05 vs. PBS, Fig. 3C), whereas fluorescein sodium scores (Fig. 3B) were significantly increased (^*^P < 0.05 vs. control, ^v^P < 0.05 vs. PBS). After 14 days of treatment, PBS-treated corneas did not show positive staining of fluorescein sodium (Fig. 3A, upper row of images), and the BUTs and tear film/epithelial damage scores were not changed (P > 0.05, Fig. 3B–E). The tear film/epithelial damage appeared in the PM_2.5_-treated group, probably due to the toxicity of the PM_2.5_ (^*^P < 0.05 vs. control, ^v^P < 0.05 vs. PBS, Fig. 3A, lower row of images, Fig. 3B–E).

### Aqueous tear volume

At day 0, there was no significant difference between the PBS- and PM_2.5_-treated groups. Compared with the vehicle group, the tear volume was decreased rapidly in the PM_2.5_-treated group after 14 days of treatment.

### Inflammation cell infiltration analysis

**Figure 4:**
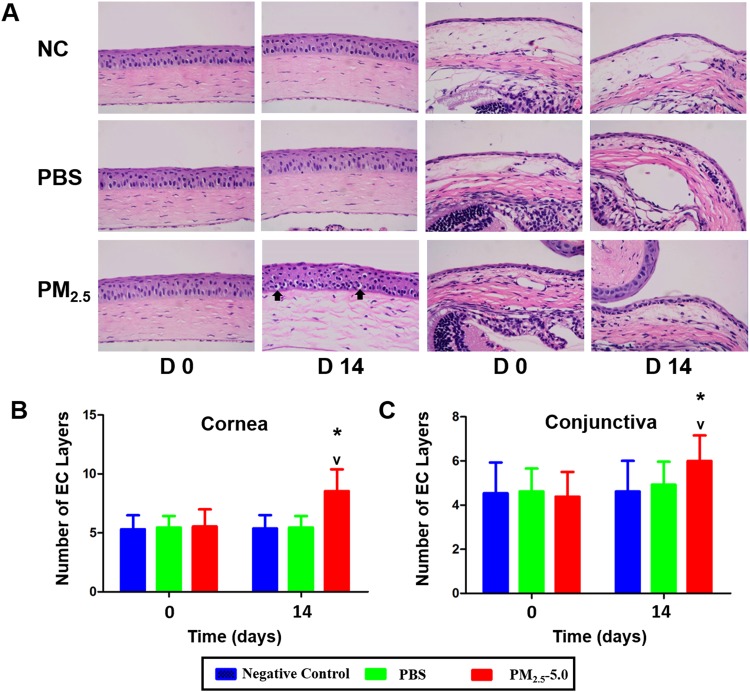
Alterations of the inflammation cell and epithelial cell after PM_2.5_ treatment. Representative images showing more inflammatory infiltration in the cornea and conjunctiva after PM_2.5_ treatment (**A**, lower row of images, black arrowheads indicate the infiltrated cells) compared with that of the control, including the NC and PBS groups (**A**, upper row and middle row of images). In the central cornea (**B**) and conjunctiva (**C**), more layers of epithelium were observed in PM_2.5_-treated eyes than in the control (P < 0.05). Each value represents the mean ± the SD, n = 5. *P < 0.05 vs. control, ^v^P < 0.05 vs. PBS.

There was a significant increase in the inflammatory index at all time points in the PM_2.5_-treated group compared with that in the PBS-treated group (^*^P < 0.05 vs. control, ^v^P < 0.05 vs. PBS, Fig. 3E). In addition, the PM_2.5_-treated group showed more infiltration of inflammatory cells in the central cornea and conjunctiva region than that in the PBS-treated group and negative control group (Fig. 4A). The number of epithelial layers in the central cornea and conjunctiva was significantly decreased in PM_2.5_-treated eyes than in control eyes after 14 days of treatment (^*^P < 0.05 vs. control, ^v^P < 0.05 vs. PBS, Fig. 4B,C).

### Inflammatory Changes in the Ocular Surface

**Figure 5:**
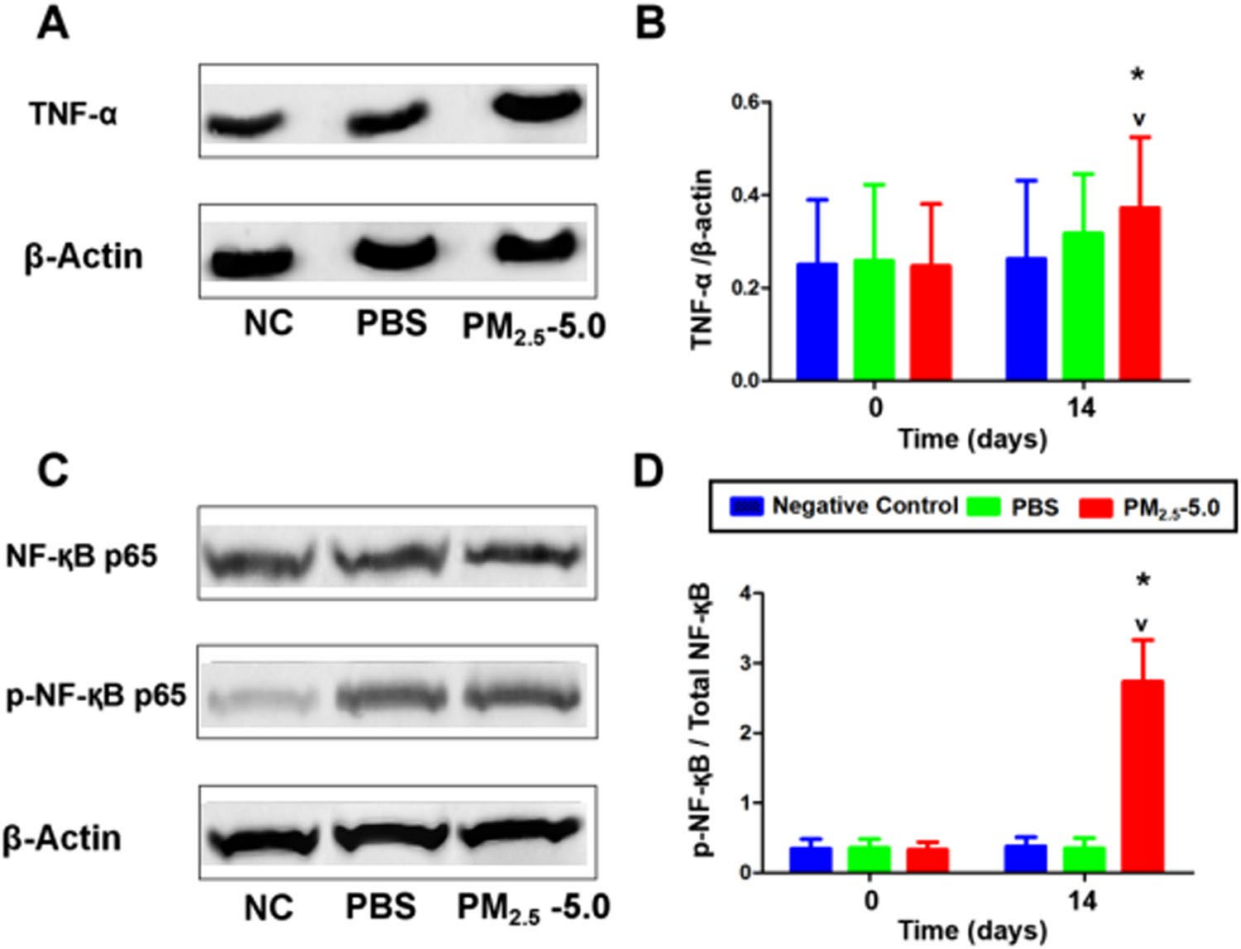
Effect of PM_2.5_ on TNF-α and NF-қB activation in the corneas evaluated by western blot analysis, with β-actin as a loading control. After treatment for 14 days, the protein level of TNF-α in PM_2.5_ cornea was upregulated and significantly higher than that in the NC and PBS groups (P < 0.05, see **A**,**B**). (**B**) Statistical analysis of the band intensity values. (**C**) Compared with the NC and PBS groups, PM_2.5_ significantly increased the phosphorylation of NF-қB (P < 0.05, see **C**,**D**). (**D**) Statistical analysis of band intensity values. Data were presented as the mean + the SD, n = 5. *P < 0.05 vs. control, ^v^P < 0.05 vs. PBS.

To investigate the mechanisms underlying PM_2.5_-induced dry eye, we analyzed the activation of NF-қB and TNF-α by western blotting. The results revealed that TNF-α protein levels were significantly higher in the PM_2.5_-treated ocular surface group than in the PBS group (^*^P < 0.05 vs. control, ^v^P < 0.05 vs. PBS, Fig. 5A,B and Supplementary figure E). After treatment for 14 days, PM_2.5_ significantly induced TNF-α expression. Similarly, the levels of NF-қB p65 and phosphorylated NF-қB p65 were markedly increased on the ocular surface by PM_2.5_ drops (^*^P < 0.05 vs. control, ^v^P < 0.05 vs. PBS, Fig. 5C,D and Supplementary figure F). Together, our results suggest that topical PM_2.5_ induces inflammation in the development of DES.

### Goblet cell density

**Figure 6:**
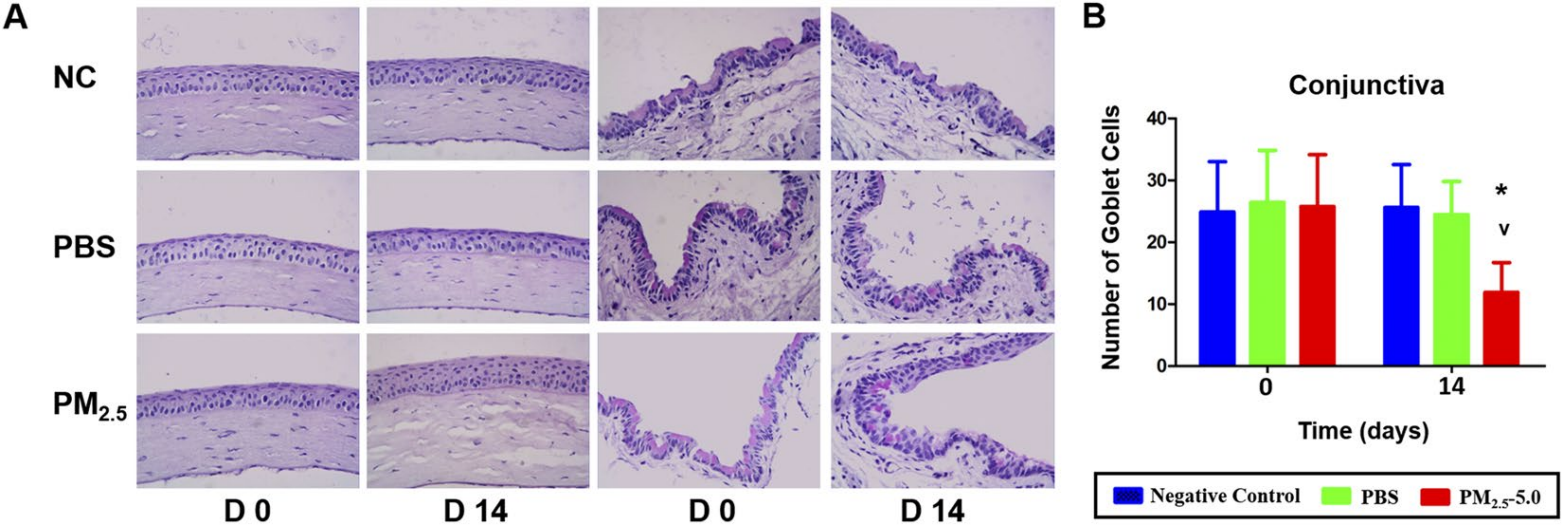
Representative images for PAS staining in the cornea and conjunctiva. No PAS-staining cells were found in the corneas of the three groups (**A**, left-hand images). PAS staining of the cornea and forniceal conjunctiva showed that, after 14 d of treatment, the goblet cells were abundantly present in the conjunctival fornix of the PBS-treated eyes but almost disappeared after PM_2.5_ treatment (A right-hand images). The average number of PAS-positive cells in the conjunctiva was significantly lower than that in PBS-treated eyes on day 14 (P < 0.05, **B**). The data are presented as the mean ± the SD, n = 5. * P < 0.05 vs. control, ^v^P < 0.05 vs. PBS.

We used PAS staining to examine the effect of PM_2.5_ on goblet cells in the cornea and conjunctiva. Interestingly, PAS-positive cells were not detected in the cornea in all groups (Fig. 6A). However, the PAS-positive cell number in the conjunctiva was significantly decreased in the PM_2.5_-treated group compared with that in the negative control and PBS treatment groups after 14 days of treatment (^*^P < 0.05 vs. control, ^v^P < 0.05 vs. PBS, Fig. 6B).

### Apoptosis and cell proliferation

**Figure 7:**
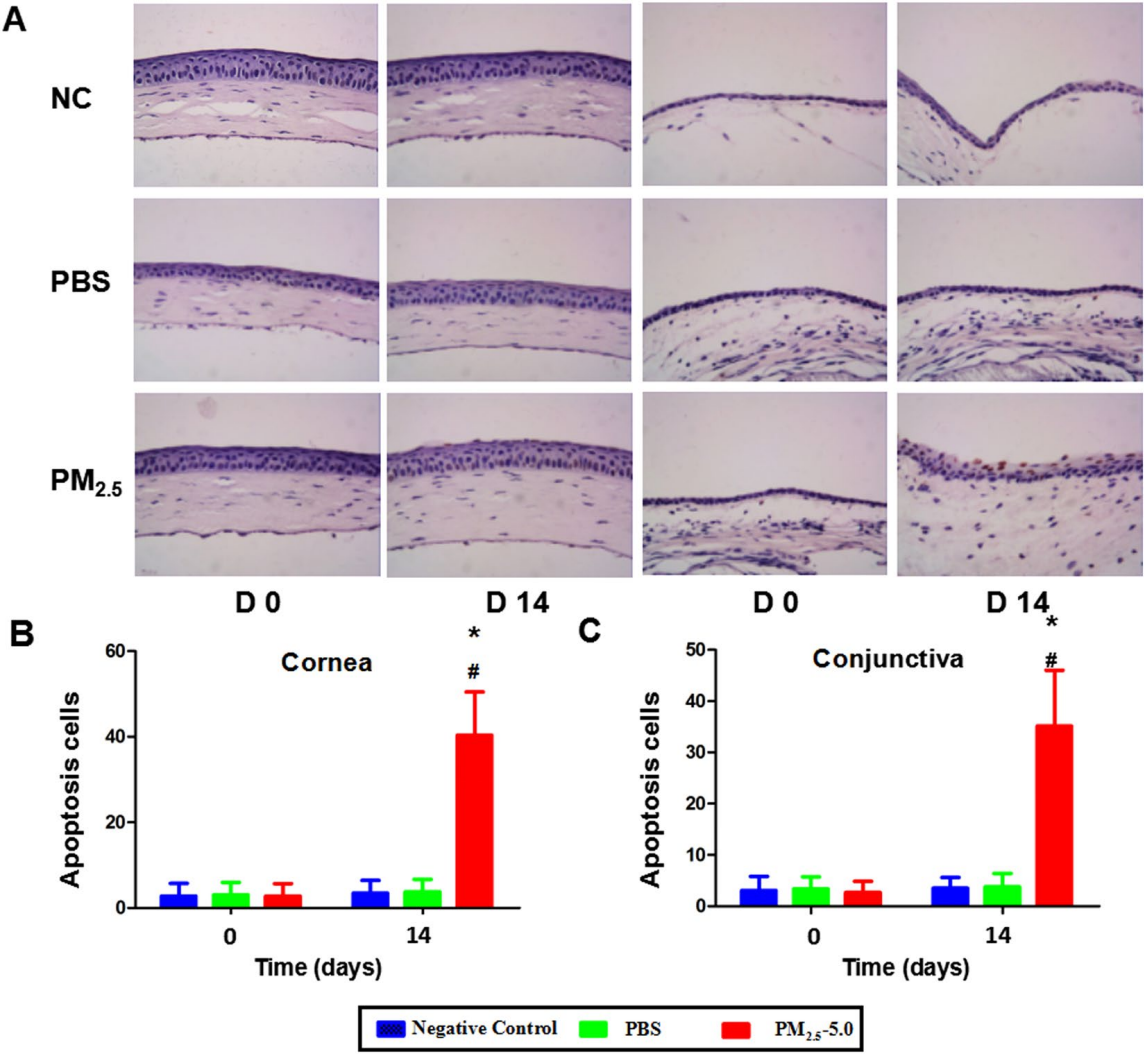
Corneal and conjunctival epithelial cell apoptosis in the NC groups, PBS-treated groups, and PM_2.5_-treated groups. Representative images for the TUNEL assay of the corneal epithelium and conjunctival epithelium on day 14 (**A**). Only a few apoptotic cells were observed in the superficial layer of the corneal and conjunctival epithelium in the control groups, while much more apoptosis was recorded in corneal and conjunctival superficial and basal epithelium after PM_2.5_ treatment (P < 0.05, **B**,**C**). The data are presented as the mean ± the SD, n = 5. *P < 0.05 vs. control, ^v^P < 0.05 vs. PBS.

**Figure 8:**
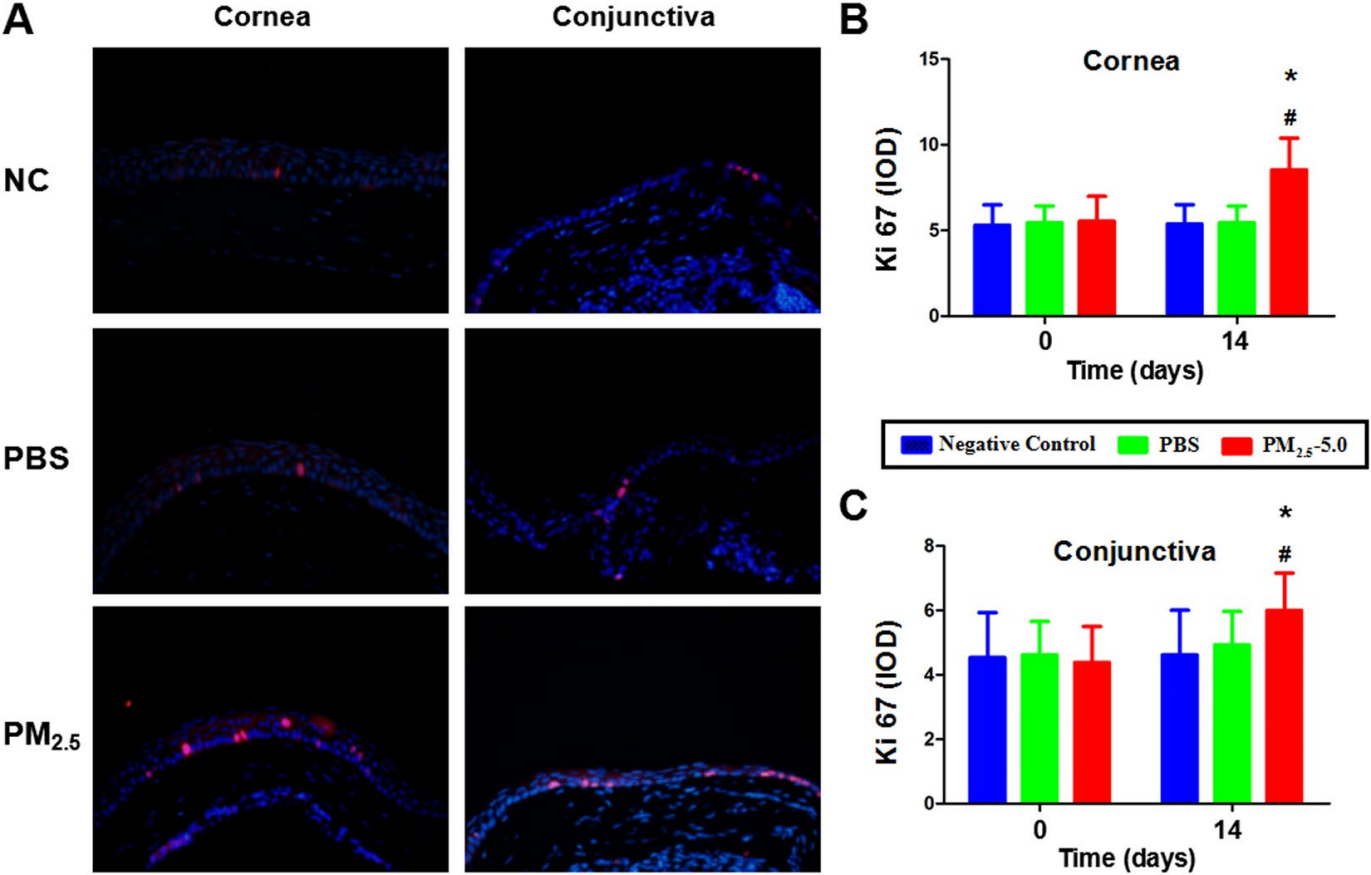
Corneal and conjunctival epithelial cell proliferation in the PBS-treated groups and PM_2.5_-treated groups. Representative images for Ki67 immunofluorescent staining of the corneal and conjunctival epithelium on day 14 (**A**). Ki67-positive cells were mainly located at the basal cell layer of corneal and conjunctival epithelium. Compared with the NC and PBS groups, a drastic increase in Ki67-positive cells was observed in both the central cornea and conjunctiva of the PM_2.5_ group after 14 days of treatment according to the cell count and IOD measurement (P < 0.05, **B**,**C**). The data are presented as the mean ± the SD, n = 5. *P < 0.05 vs. control, ^v^P < 0.05 vs. PBS.

The TUNEL assay showed that apoptosis was induced in the corneal superficial and basal epithelium but not in the stroma in the PM_2.5_-treated group, while few apoptotic cells were observed in the corneal epithelium of the PBS-treated group and negative control group (^*^P < 0.05 vs. control, ^v^P < 0.05 vs. PBS, Fig. 7A–C). Compared with the control groups, the immunostaining of Ki67 revealed a drastic increase in Ki67-positive cells in both the central cornea and conjunctiva of the PM_2.5_ group after 14 days of treatment (^*^P < 0.05 vs. control, ^v^P < 0.05 vs. PBS, Fig. 8A–C), and Ki67-positive cells were mainly located at the basal cell layer of the cornea and conjunctiva.

### Corneal Epithelial Ultrastructural changes

**Figure 9:**
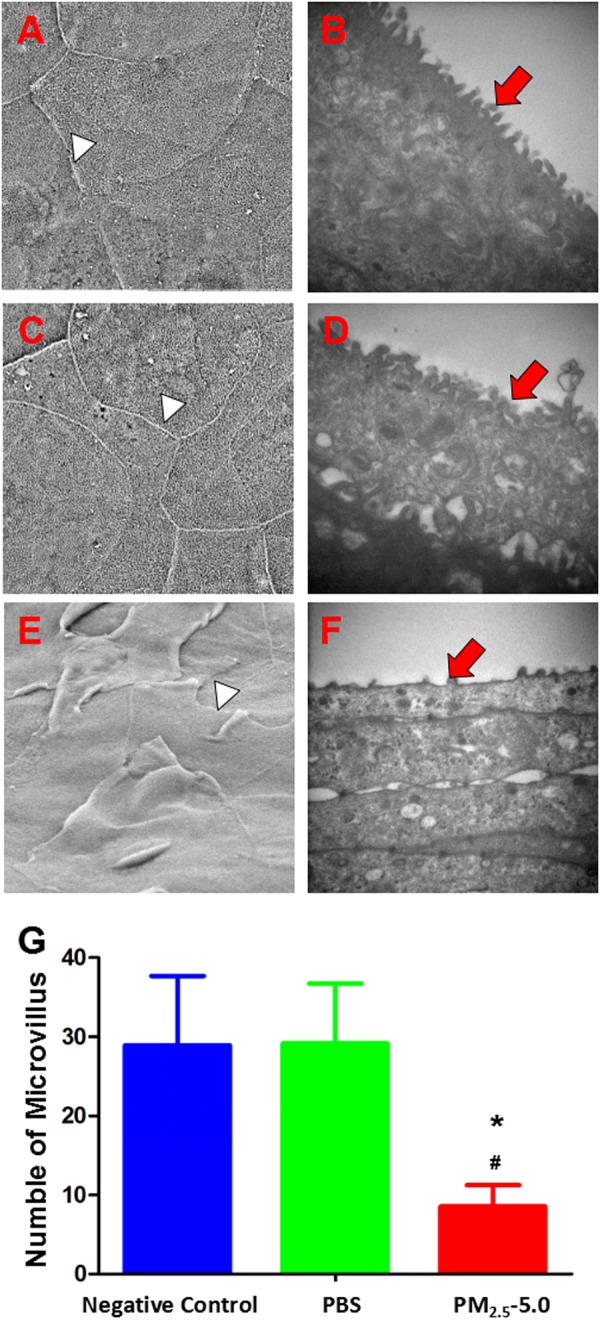
Scanning and transmission electron microscopy images showing the ultrastructure of the corneal epithelium in the negative control group, PBS-treated group and PM_2.5_-treated groups on day 14. In NC and PBS-treated mice, the intact junction existed clearly (**A**,**C**, white arrow heads), and epithelial microvilli were extended digitately and arranged neatly (**B** and **D**, red arrow heads) (**A**–**D**). By contrast, the disrupted junction (**E**, white arrow head) and disordered corneal epithelial microvilli (**F**, red arrow head) were observed in PM_2.5_-treated. Only a few microvilli were observed in the superficial layer of the corneal epithelium in the PM_2.5_ treatment group, while many more microvilli were recorded in the corneal epithelium after PBS treatment (**G**, P < 0.05). The data are presented as the mean ± the SD, n = 3. *P < 0.05 vs. control, ^v^P < 0.05 vs. PBS.

Corneal epithelium cells have many neatly arranged microvilli and microfolds extending outwards in the negative control (Fig. 9A,B). The corneal epithelium was intact and organized in groups A and B (Fig. 9A–D). By contrast, the epithelial cells were deformed in the cornea of the PM_2.5_-treated eyes (Fig. 9E,F). After PBS treatment for 14 days in group A, transmission electron microscopy revealed enriched regularly arranged microvilli extending from surface epithelial cells (Fig. 9B, upper line). By contrast, after 14 days of PM_2.5_ treatment in group C, the number of corneal epithelial microvilli (Fig. 9G) was drastically reduced, the morphology of the microvilli was significantly different to that in the other two groups (all ^*^P < 0.05 vs. control, ^v^P < 0.05 vs. PBS), and most of the microvilli were much shorter and disorganized (Fig. 9E,F).

### Apoptosis of lacrimal glands

**Figure 10:**
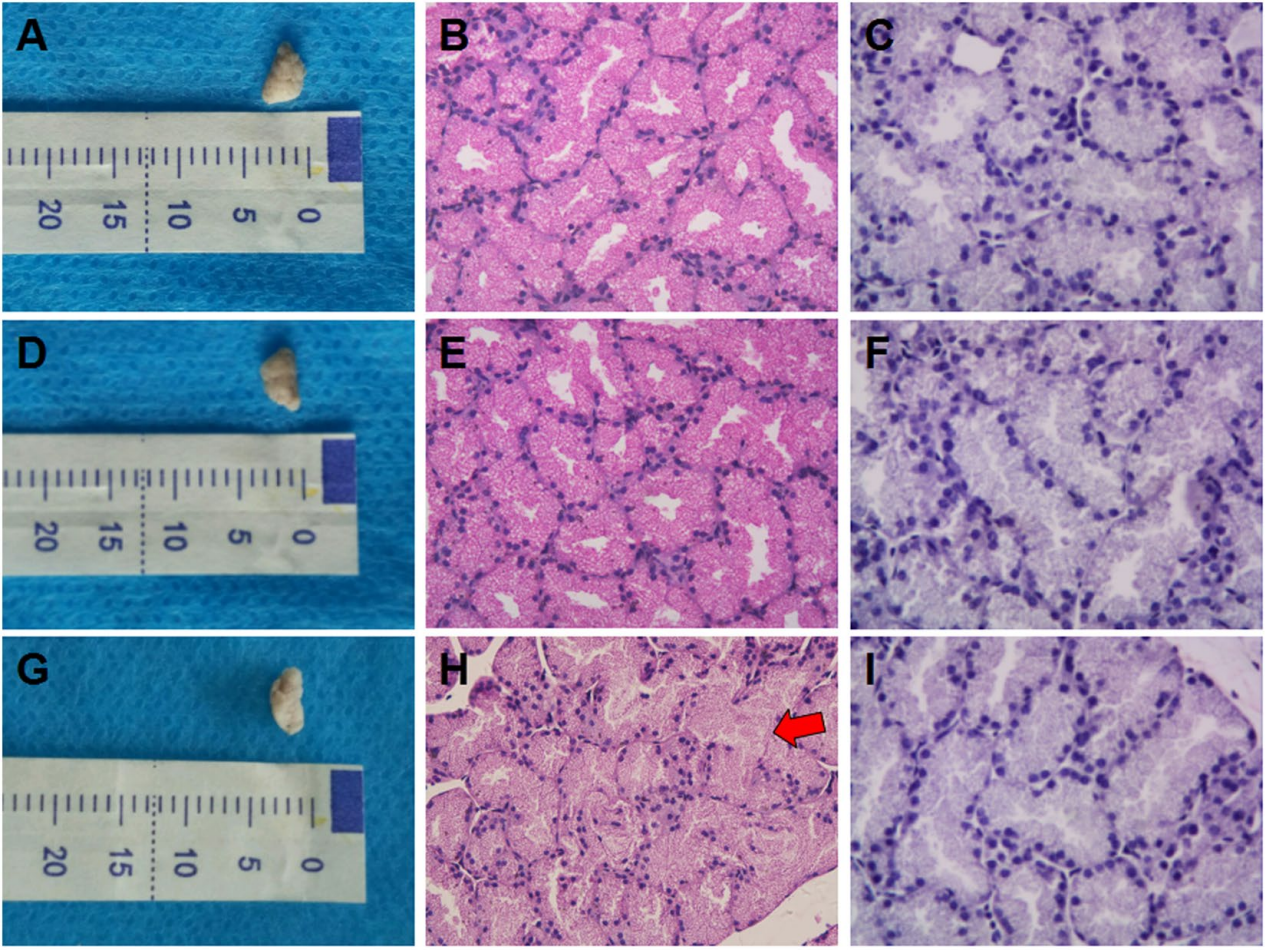
Images of lacrimal glands from all three groups (n = 3 in each group**)**. Lacrimal glands from the NC groups (**A**) and PBS-treated groups (**D**) maintained the same volume and color. By contrast, lacrimal glands from the PM_2.5_-treated groups (**G**) were shrunken and became lighter. (**H**,**E**) Staining showed similar images of the maintenance of the complete structure of the lobe, duct, and acinar of lacrimal glands in both the NC and PBS-treated groups, and no inflammatory cells were observed (**B**,**E**). However, in the PM_2.5_-treated groups, irregularly arranged lobes were found (**H**, red arrow head). TUNEL assay analysis detected no apoptotic bodies among all three groups (**C**,**F**,**I**).

Lacrimal glands from the NC groups (Fig. 10A) and PBS-treated groups (Fig. 10D) were similar in volume and color. By contrast, lacrimal glands from PM_2.5_-treated groups (Fig. 10G) were shrunken and lighter in color. H&E staining revealed that the lobe, duct, and acinar of lacrimal glands were maintained properly in both the NC and PBS-treated groups, and no inflammatory cells were observed (Fig. 10B,E). However, irregularly arranged lobes were often found in the PM_2.5_-treated lacrimal glands (Fig. 10H). There were no apoptotic bodies observed by TUNEL assay analysis in all three groups (Fig. 10C,F,I).

## Discussion

To the best of our knowledge, this is the first study to evaluate the effects of air pollutant PM_2.5_ in inducing dry eye in mice. PM_2.5_, as one of the main components of atmospheric pollutants, has aroused widespread concern with regard to its impact on health. The effect of particles on the human body is related to the diameter of the particle itself^29^. Normally, large-diameter particles will be filtered through nasal cilia and mucus and cannot pass through the nose and throat. Particles with a diameter of less than 10 µm can infiltrate the lungs and bronchial alveolar structures^30^. Particles with a diameter of less than 2.5 µm have a greater penetration potential. They may infiltrate the fine bronchial wall and interfere with gas exchange in the lungs^31^. These particles can eventually enter the blood vessels, where they might impact other parts of the body through blood circulation^32^, making PM_2.5_ more detrimental. BURNETT *et al*. surveyed eight cities in Canada and found that smaller particles cause more serious damage to the human body^33^. In summary, atmospheric particulate matter increases the occurrence rate of mortality, asthma, atherosclerosis, and diabetes^34–37^.

The eye is in direct contact with the outside world, so changes in the external environment will have a major impact on the ocular microenvironment^38,39^. Dry eye is a common disease on the eye surface, and environmental factors are one of the main causes^40^. Normally, the eye surface is covered with tear film, and the stability of tear film is crucial for maintaining ocular health. The stability of tear film depends on the normal components of each layer in the tear film and normal tear dynamics^41^. Environmental factors can affect the composition of the lacrimal film, and it can also affect tear dynamics. In our experiment, we found that BUT and the corneal FL score were increased significantly, while SIT was reduced in the group treated with PM_2.5_ for 14 days compared with the control treatment group and negative control group. These results indicate that the stability of the tear film is impaired, which will cause further damage to the cornea. Tiger red staining and fluorescein sodium staining showed corneal epithelial defects in the PM_2.5_-treatment group. H&E staining indicated that the number of corneal and conjunctival epithelial layers was increased drastically in the PM_2.5_-treatment group, and the arrangement was disorganized. We also found that lacrimal gland epithelial cells were reduced after PM_2.5_ treatment, while no apoptosis cells were observed. Under the electron microscope, we observed finger-like projections of microvilli of the corneal epithelium. Similar to the negative control group, microvilli were increased in number and were arranged neatly after 14 days of PBS eye drops. By contrast, the corneal epithelial microvilli were reduced in number and became shorter and disordered after treatment with PM_2.5_ for 14 days. Mucus protein secreted by goblet cells is one of the components of tear film and a very important factor in maintaining the stability of tear film^42^. In dry eye patients, decreased conjunctival goblet cells result in decreased secretion of mucin, thereby reducing the stability of tear film^43^. This change was verified in our experiments. Similar to those with dry eye, conjunctival goblet cells were significantly fewer in PM_2.5_-treated eyes compared with the PBS-treated group and negative control.

PM_2.5_ not only affects the stability of tear film but also introduces many toxic and harmful substances^44^. *In vitro* results have suggested that PM_2,5_ can directly impact corneal cellular health because exposure to PM_2,5_ affects HCE cell viability. With the increase in the dosage of PM_2.5_, the survival rate of HCE decreases. According to our *in vitro* experiments, we explored the *in vivo* effects of PM_2.5_ of different concentrations. We found that 0.5-mg/ml PM_2.5_ administration did not cause any changes in tear secretion as shown by *in situ* staining (data not shown). It is possible that PM_2.5_ eye drops in animal studies are diluted quickly due to tear secretion and blinking. By contrast, 10 mg/ml PM_2.5_ was too irritating in mice. Corneal angiogenesis occurred at approximately 2 weeks (data not shown). Thus, we chose 5 mg/ml as the optimal PM_2.5_ eye drop concentration to induce dry eye syndrome in the present study. It would be interesting to investigate the ocular surface damage induced by different PM_2.5_ concentrations in future toxicity studies. In addition, the uptake of 2.5-µm diameter latex beads by HCECs suggests that PM_2.5_ can be phagocytized into HCECs and intoxicate cells. The results of the scratch experiment showed that PM_2.5_ could inhibit the migration and proliferation of HCE. These may be additional mechanisms of PM_2.5_-induced dry eye.

A recent study reported that NF-κB can regulate the gene expression of various cytokines and adhesion molecules involved in the inflammatory response and is closely related to the occurrence of inflammation^45,46^. In addition to its role in inflammation, the NF-κB pathway also affects cell proliferation and anti-apoptosis^47^. Moreover, the activation of the NF-κB pathway includes the involvement of upstream and downstream genes, and the interaction between apoptosis and anti-apoptotic genes^46^.

Existing studies have found that genes involved in the inflammatory response of dry eye are mostly target genes of NF-κB. In a rabbit dry eye model, there is increased expression of NF-κB P65 in the cornea, conjunctiva, and lacrimal gland tissue, indicating the activation of NF-κB. Activated NF-κB enters the nucleus, where it binds to κB (GGGACTTTCC) of the NOS2 target gene promoter to induce transcription and promote the synthesis of target genes (e.g., TNF). Therefore, the activation of NF-κB may be one of the initiation mechanisms of dry eye^48^. Apoptosis is an important mechanism in the development of dry eye. The apoptosis index of epithelial cells in dry eye is increased^49^. Dry eye is an immune disease, and apoptosis is involved in the development of immune cells, immune regulation, immune effect, and many other physiological and pathological processes^50^. In our experiment, after PM_2.5_ treatment, the apoptotic cells in the corneal epithelial cells were significantly increased compared with those in the PBS treatment group and negative control group. Western blotting revealed that the PM_2.5_-treatment group showed higher levels of pNF-κB expression than the other two groups.

The ratio of pNF-κB to pan NF-κB was higher than that in the two control groups, indicating that NF-κB is activated, which is further validated by the increase in TNF-α expression. Thus, we hypothesize that the mechanism of PM_2.5_-induced dry eye may be mediated by the activation of NF-κB. It will be of great value to verify the potential relationship between NF-κB activation and the progression of dry eye, which might promote the development of dry eye therapy.

One major caveat of the study is that the environmental exposure of PM2.5 may be different from direct PM2.5 topical administration. We will modify the concentration and the mode of contact in future studies to better simulate PM2.5 environmental exposure.

## Electronic supplementary material

Supplementary Information
