# Supplementary material for: Air pollutant particulate matter 2.5 induces dry eye syndrome in mice
Source: Sci Rep. 2018 Dec 13;8:17828. doi: 10.1038/s41598-018-36181-x (PMC6292905; doi:10.1038/s41598-018-36181-x)
Supplement: Supplementary file 1 — Supplementary Information [file 41598_2018_36181_MOESM1_ESM.docx]

**Air pollutant particulate matter 2.5 induces dry eye syndrome in mice**

**Running title: PM2.5 induces DES**

Gang Tan,^1,2*^ Juan Li,^3*^ Qichen Yang,^4^ Anhua Wu,^2^ Dong-Yi Qu,^5^ Yahong Wang,^6^ Lei Ye,^1^ Jing Bao,^1^ Yi Shao^1^

^1^Department of Ophthalmology, the First Affiliated Hospital of Nanchang University, Nanchang 330006, Jiangxi Province, China;

^2^Department of Ophthalmology, the First Affiliated Hospital of University of South China, Hengyang, 421001, Hunan Province, China;

^3^Department of Ophthalmology, the Fourth Hospital of Xi'an, Xi’an 710004, Shanxi Province, China;

^4^Eye Institute of Xiamen University, Xiamen, Fujian 361102, China;

^5^Department of ophthalmology, Haidian maternal & child health hospital, Beijing 100080, China;

^6^Environmental Monitoring Station of Xi'an City, Xi’an 710054, Shanxi Province, China;

*These authors have contributed equally to this work.

Correspondence: Yi Shao, Department of ophthalmology, The First Affiliated Hospital of Nanchang University, No 17, Yongwaizheng Street, DongHu District, Nanchang 330006, Jiangxi, China. Email: freebee99@163.com

**Competing financial interests statement**

This was not an industry supported study. The authors report no conflicts of interest in this work

**Acknowledgments**

This study was supported by National Natural Science Foundation of China (No: 81660158, 81400372, 81400424); Science and Technology Research and Development Project of Shanxi Province (2014K11-03-07-04)；Innovative Talents Promotion Project of Shanxi Province(2017); Youth Science Foundation of Jiangxi Province (No: 20151BAB215016); Key Research Development Foundation of Jiangxi Province(No: 20151BBG70223); Key Education Department Foundation of Jiangxi Province (No: GJJ160020); Health Development Planning Commission Science Foundation of Jiangxi Province(No: 20175115)

**Author contributions:**

G.T., J.L., Q.C.Y. and Y.S. conceived the research. G.T., J.L. and Y.S. developed the experimental setup. G.T., J.L., Q.C.Y. and A.H.W. performed the experiments. Q.C.Y., D.Y.Q., Y.H.W. and L.Y. analyzed the data. The manuscript was prepared by G.T., J.L., Q.C.Y., J.B. and Y.S. All authors discussed the results and commented the manuscript.


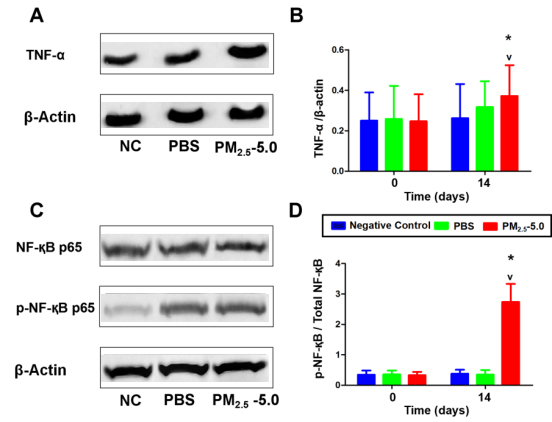


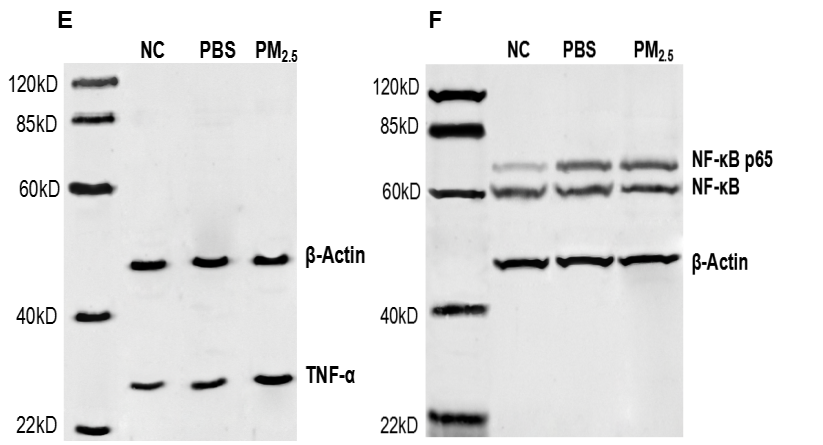


Figure **E** is the original full gels of figure A,figure **F** is the original full gels of figure **C**
